# Protecting liver health with microbial-derived succinylated bile acids

**DOI:** 10.1093/lifemeta/loae023

**Published:** 2024-06-13

**Authors:** Hadrien Demagny, Alessia Perino, Kristina Schoonjans

**Affiliations:** Laboratory of Metabolic Signaling, Institute of Bioengineering, School of Life Sciences, École Polytechnique Fédérale de Lausanne, 1015 Lausanne, Switzerland; Laboratory of Metabolic Signaling, Institute of Bioengineering, School of Life Sciences, École Polytechnique Fédérale de Lausanne, 1015 Lausanne, Switzerland; Laboratory of Metabolic Signaling, Institute of Bioengineering, School of Life Sciences, École Polytechnique Fédérale de Lausanne, 1015 Lausanne, Switzerland

**The gut microbiome produces a plethora of metabolites that influence host metabolism. In a recent article published in *Cell*, Nie and colleagues reveal that 3-succinylated cholic acid, a microbe-derived bile acid, protects against metabolic dysfunction-associated liver disease. The authors show that an increased abundance of the beneficial commensal microbe *Akkermansia muciniphila*, rather than enhanced bile acid signaling, drives this protective phenotype.**

The gut is a complex ecosystem housing a diverse array of microorganisms closely connected to host health and disease. As part of its symbiotic relationship with the host, the microbiome transforms diet- and host-derived molecules into metabolites that serve signaling functions. Among these signaling molecules are bile acids (BAs). In humans, the liver synthesizes two BAs from cholesterol: cholic acid (CA) and chenodeoxycholic acid (CDCA). In addition, the rodent liver can convert CDCA into muricholic acids (αMCA and βMCA). These BAs, derived from the host, are known as primary BAs. Before being released in bile, they are conjugated with either glycine or taurine in humans or taurine in rodents. As a result, primary BAs entering the biliary system and eventually reaching the proximal small intestine are essentially present as conjugated species. The small fraction of primary BAs that escapes reabsorption in the distal ileum enters the microbe-dense colon and fuels a complex series of bacteria-driven reactions. Apart from the removal of the amino acid moiety of conjugated BAs, defined as deconjugation, three additional reaction types are known to be catalyzed by microbial enzymes and lead to the formation of so-called secondary BAs. These reactions include removal (dehydroxylation), oxidation (dehydrogenation), and epimerization of nuclear hydroxyl groups [1]. Deconjugation of BAs, in particular, is a prerequisite for 7α-dehydroxylation of CA and CDCA to form deoxycholic acid (DCA) and lithocholic acid (LCA), respectively, and is catalyzed by bacterial species with bile salt hydrolase (BSH) activity. In 2020, a fifth type of reaction was first reported by Quinn *et al*. [2]. Using an advanced mass spectrometric approach to identify bacterial metabolites across the entire body in mice, the authors discovered a series of atypical conjugates of CA with leucine, phenylalanine, and tyrosine. These novel BA conjugates were shown to be of microbial origin and readily detected in the intestines of specific pathogen-free (SPF) but not germ-free mice [2]. Since then, our understanding of the BA spectrum produced by gut microbes has greatly expanded. This year alone, the discovery of new BAs and BA-modifying microbes has remarkably increased, fueled by the use of advanced omics technologies, which have opened new research avenues [1]. Techniques such as high-throughput sequencing, mass spectrometry, and bioinformatics analyses have been particularly instrumental in identifying novel BAs and understanding their biosynthetic pathways. The integration of these technologies into BA research has not only accelerated the pace of discovery but also enhanced the resolution at which we can study these molecules.

Although primary and secondary BAs were initially only recognized for their role in lipid digestion, subsequent research revealed that these cholesterol derivatives have a much more complex role in human physiology [3]. Beyond their digestive functions, BAs circulate throughout the body, acting as signaling molecules that influence systemic metabolism. This signaling occurs through the activation of specific receptors, such as the farnesoid X receptor (FXR) and the G protein-coupled bile acid receptor 1 (GPBAR1, also known as Takeda G protein-coupled receptor 5 [TGR5]). Additionally, BAs exhibit selective antimicrobial activity, which shapes the gut microbiota. The intricate interactions between BAs, gut microbes, and host BA receptors significantly impact host (patho)physiology, highlighting the multifaceted roles of BAs in maintaining health and contributing to disease processes.

In a recent issue of *Cell*, Nie and colleagues explored the complex relationship between BAs and the microbiome in the development of metabolic dysfunction-associated fatty liver disease (MAFLD) [4]. MAFLD is characterized by excess fat accumulation or steatosis in the liver and is primarily associated with metabolic conditions such as obesity, type 2 diabetes, and insulin resistance. If untreated, MAFLD can progress from steatosis to metabolic dysfunction-associated steatohepatitis (MASH), potentially leading to liver fibrosis, cirrhosis, and even liver failure or cancer. In this study, Nie and colleagues utilized a novel click-chemistry enrichment technique involving CA to create an alkyne-tagged probe (alkCA) for enhanced detection in liquid chromatography-tandem mass spectrometry. Incubating alkCA with microbial communities from human stool samples produced several previously unidentified CA derivatives, including 3-succinylated CA (3-sucCA). The authors discovered that bacteria require the presence of a β-lactamase enzyme, which they referred to as BA acyl synthetase for succinyl (BAS-suc), to carry out the succinylation reaction and identified *Bacteroides uniformis* (*B. uniformis*) as an endogenous producer of 3-sucCA. In addition to characterizing the biochemical reactions linked to 3-sucCA, the authors also focused on identifying its role in host (patho)physiology. Interestingly, 3-sucCA levels decreased as MAFLD advanced to MASH and showed an inverse correlation with MAFLD severity in humans. Oral administration of 3-sucCA in murine models of MASH led to reduced liver steatosis, inflammation, and fibrosis. Of note, these beneficial effects were not linked to the activation of known BA receptors like TGR5 and FXR, as might be expected. Instead, they were associated with changes in the gut microbiota. Specifically, 3-sucCA was found to promote the growth of *Akkermansia muciniphila* (*A. muciniphila*), a microbe known for enhancing intestinal barrier integrity. This gut health improvement was essential for the liver-protective effects observed, revealing a new therapeutic mechanism for secondary BAs that operates independently of canonical BA signaling pathways (Fig. 1).

**Figure 1:**
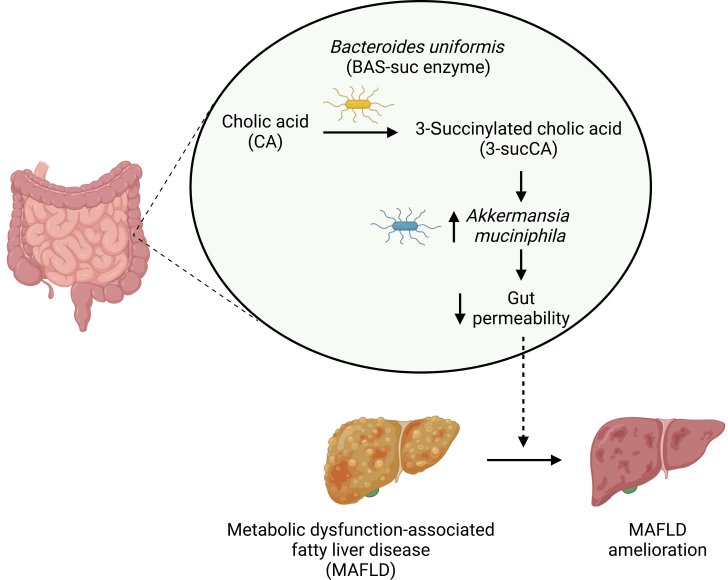
*B. uniformis* converts CA into 3-sucCA, which fosters the growth of the beneficial microbe *A. muciniphila*. This process reduces gut permeability, thereby alleviating MAFLD. The figure is created with Biorender.com.

*A. muciniphila* is an abundant gut bacterium that has attracted considerable scientific interest due to its potential therapeutic effects on metabolic disorders such as obesity, type 2 diabetes, and MAFLD [5]. This bacterium, which naturally inhabits the human gastrointestinal tract, is essential for maintaining the integrity of the intestinal mucosal layer. Studies indicate that *A. muciniphila* enhances gut barrier function and mitigates liver inflammation by downregulating hepatic Toll-like receptor 2. This downregulation reduces the pro-inflammatory responses driven by γδT and γδT17 cells and promotes a shift in macrophage polarization towards the anti-inflammatory M2 phenotype, thereby alleviating MAFLD and MASH in murine models [6]. MAFLD is frequently associated with dysbiosis of the gut microbiota, leading to significant health issues. Dysbiosis refers to an imbalance in the microbial communities residing in the gut, which can result in the proliferation of harmful bacteria and a reduction in beneficial bacteria. This imbalance often triggers an inflammatory response, compromising the integrity of the intestinal barrier. When the intestinal barrier is weakened, it becomes more permeable, allowing harmful microbial by-products, such as endotoxins, to enter the bloodstream. These toxic substances can travel to the liver, where they exacerbate liver inflammation and contribute to the progression of MAFLD.

Given the detrimental ramifications of a disrupted gut barrier, targeting the microbiome and BA pathways, as proposed by Nie and colleagues, constitutes a promising strategy to maintain the integrity of the intestinal mucosal layer and mitigate the progression of MAFLD. This approach would leverage the modulation of gut microbiota and BA dynamics without the need to directly alter hepatic BA metabolism, potentially offering a novel and effective treatment strategy for MAFLD. Early efforts to modify BA signaling pathways focused on using FXR agonists, particularly obeticholic acid (OCA), a semi-synthetic BA analog tested for managing MAFLD/MASH [7]. More recent strategies have investigated the therapeutic potential of gut microbiota-modified BAs, such as hyodeoxycholic acid (HDCA). HDCA inversely correlates with MAFLD severity and, when administered to mice, mitigates disease progression. Like 3-sucCA, HDCA does not reach hepatic cells but enhances liver health by modulating the gut-liver axis [8]. Additionally, other indirect BA signaling interventions, like the glycine-based tripeptide DT-109, alleviate MASH in rodents and non-human primates by altering gut microbiota composition and reducing the production of the highly hydrophobic secondary LCA species [9].

Building on these studies, Nie and colleagues demonstrated that administering 3-sucCA could also provide therapeutic benefits, although further research is needed to fully elucidate the underlying mechanisms of these protective effects. 3-sucCA appears to modulate the *B. uniformis/A. muciniphila* axis, both *in vitro* and in animal models. Yet, it is plausible that 3-sucCA may also influence other, potentially unidentified, bacterial species. Such interactions could be revealed using advanced omics approaches beyond traditional 16S rRNA gene amplicon sequencing, followed by thorough *in vitro* investigations for validation. Another outstanding matter concerns the bacterial import of (de)conjugated CA and the release of re-conjugated 3-sucCA. Carrier proteins are likely necessary for the bidirectional transport of (un)conjugated BAs across the bacterial outer and inner membranes. For example, bacteria such as *Clostridium scindens*, which can perform 7α-dehydroxylation of BAs, express a membrane transporter (*baiG*) that facilitates the energy-dependent uptake of preferably unconjugated primary BAs [10]. Identifying and cloning the complete set of proteins involved in the formation and release of 3-sucCA—beyond BAS-suc—will provide further insights into their substrate specificity, cofactors, and bioenergetic requirements. This understanding will enhance our knowledge of the mechanisms underlying BA modification and transport within bacterial cells. Additionally, while 3-sucCA does not seem to activate the most extensively studied BA receptors, it may engage other, less well-characterized receptors expressed in host gut cells. Indeed, over the years, several other receptors have been proposed to drive part of the immunomodulatory actions of BAs in the liver and intestine, including the sphingosine-1-phosphate receptor 2 (S1PR2), the pregnane X receptor (PXR), the constitutive androstane receptor (CAR), the vitamin D receptor (VDR), and the retinoic acid-related orphan receptor γt (RORγt) [3]. Further elucidation of the intricate mechanisms of 3-sucCA’s interactions with both microbial and host systems, including its transport, receptor engagement, and potential influence on unidentified bacterial species, will advance our understanding of its therapeutic potential.

A remaining conundrum, applicable not only to 3-sucCA but also to other low-abundance secondary BAs, pertains to the stability and bioavailability of these microbial products. This issue is particularly significant given the known susceptibility of the N-amidate bond to enzymatic cleavage in BAs conjugated with amino acids other than glycine or taurine [11]. Indeed, it is believed that the selection of taurine and glycine as amino acid substrates in the hepatic N-amidation of bile salts is less about the specificity of the hepatic enzyme bile acid CoA: amino acid N-acyltransferase (BAAT) and more about the resistance of their respective conjugates to degradation by host enzymes encountered during their enterohepatic circulation, such as carboxypeptidases in pancreatic juice and (carboxy) peptidases in the small intestine [11]. This understanding emerged from studies with synthetic bile salt conjugates, which were susceptible to cleavage by pancreatic carboxypeptidases unless conjugated with glycine or taurine. Conjugates with these particular amino acids are largely resistant to carboxypeptidase-mediated enzymatic degradation. It is unlikely that carboxypeptidases can remove the succinyl group from 3-sucCA, but it remains to be tested whether 3-sucCA is deconjugated *in vitro* by host enzymes catalyzing the removal of the succinyl moiety. Answering these questions will help elucidate the role of endogenous 3-sucCA in modulating host physiology and its distribution across various biological compartments. The gut-restricted nature of 3-sucCA is particularly intriguing and could be due to impaired colonic absorption caused by the absence of specific transporters, or due to deconjugation by circulating enzymes once it enters the systemic circulation. This question is all the more important given that most newly identified microbe-derived BAs, including 3-sucCA, exist in trace concentrations, complicating both their detection and the elucidation of their functions within the host. The low endogenous levels of novel secondary BAs can result from a low *de novo* synthesis rate or a very short half-life due to rapid deconjugation by host enzymes or microbial BSH. In turn, the low abundance of these novel secondary BAs suggests that they may participate in more nuanced or highly specific biological processes, potentially acting through distinct, yet-to-be-discovered pathways or mechanisms. Another possible explanation for the low levels of 3-sucCA, left unaddressed by Nie and colleagues due to the lack of nutritional status specification during BA quantification, is whether 3-sucCA levels fluctuate across various body compartments based on dietary intake. This is a critical area of inquiry because dietary intake is known to influence BA metabolism significantly.

In conclusion, this study highlights the therapeutic potential of microbiome-tailored BA modifications in the development of MAFLD. However, while these approaches show promise for managing various diseases, their clinical application is still emerging. More preclinical studies are needed to understand the dynamics of biotherapeutic agents within the complex gut microbiota.

## References

[1] Ridlon JM, Gaskins HR. Nat Rev Gastroenterol Hepatol 2024;21:348–64. 38383804 10.1038/s41575-024-00896-2 PMC11558780

[2] Quinn RA, Melnik AV, Vrbanac A et al. Nature 2020;579:123–9. 32103176 10.1038/s41586-020-2047-9 PMC7252668

[3] Perino A, Demagny H, Velazquez-Villegas LA et al. Physiol Rev 2021;101:683–731. 32790577 10.1152/physrev.00049.2019

[4] Nie Q, Luo X, Wang K et al. Cell 2024;187:2717–34.e33. 38653239 10.1016/j.cell.2024.03.034

[5] Cani PD, Depommier C, Derrien M et al. Nat Rev Gastroenterol Hepatol 2022;19:682. 35739354 10.1038/s41575-022-00650-6

[6] Han Y, Ling Q, Wu L et al. Gut Microbes 2023;15:2221485. 37345844 10.1080/19490976.2023.2221485 PMC10288935

[7] Younossi ZM, Stepanova M, Nader F et al. Clin Gastroenterol Hepatol 2022;20:2050–8.e12. 34274514 10.1016/j.cgh.2021.07.020

[8] Kuang J, Wang J, Li Y et al. Cell Metab 2023;35:1752–66.e8. 37591244 10.1016/j.cmet.2023.07.011

[9] Qu P, Rom O, Li K et al. Cell Metab 2023;35:742–57.e10. 37040763 10.1016/j.cmet.2023.03.013

[10] Mallonee DH, Hylemon PB. J Bacteriol 1996;178:7053–8. 8955384 10.1128/jb.178.24.7053-7058.1996 PMC178615

[11] Huijghebaert SM, Hofmann AF. Gastroenterology 1986;90:306–15. 2867000 10.1016/0016-5085(86)90925-x

